# Draft genome sequence of Karnal bunt pathogen (*Tilletia indica*) of wheat provides insights into the pathogenic mechanisms of quarantined fungus

**DOI:** 10.1371/journal.pone.0171323

**Published:** 2017-02-02

**Authors:** Anil Kumar, Vishakha Pandey, Manoj Singh, Dinesh Pandey, M. S. Saharan, Soma S. Marla

**Affiliations:** 1 Department of Molecular biology and Genetic Engineering, G.B. Pant University of Agriculture and Technology, Pantnagar, Uttarakhand, India; 2 Division of Plant Pathology, Indian Agricultural Research Institute, New Delhi, India; 3 Division of Genomic Resources, National Bureau of Plant Genetic Resources, New Delhi, India; Murdoch University, AUSTRALIA

## Abstract

Karnal bunt disease in wheat is caused by hemibiotrophic fungus, *Tilletia indica* that has been placed as quarantine pest in more than 70 countries. Despite its economic importance, little knowledge about the molecular components of fungal pathogenesis is known. In this study, first time the genome sequence of *T*. *indica* has been deciphered for unraveling the effectors’ functions of molecular pathogenesis of Karnal bunt disease. The *T*. *indica* genome was sequenced employing hybrid approach of PacBio Single Molecule Real Time (SMRT) and Illumina HiSEQ 2000 sequencing platforms. The genome was assembled into 10,957 contigs (N50 contig length 3 kb) with total size of 26.7 Mb and GC content of 53.99%. The number of predicted putative genes were 11,535, which were annotated with Gene Ontology databases. Functional annotation of Karnal bunt pathogen genome and classification of identified effectors into protein families revealed interesting functions related to pathogenesis. Search for effectors’ genes using pathogen host interaction database identified 135 genes. The *T*. *indica* genome sequence and putative genes involved in molecular pathogenesis would further help in devising novel and effective disease management strategies including development of resistant wheat genotypes, novel biomarkers for pathogen detection and new targets for fungicide development.

## Introduction

Wheat belongs to genus *Triticum* of an economically important family Poaceae. In 2015, world production of wheat was 732 million tons, making it the second most produced cereal after maize (972 million tons) (www.igc.int). According to Food and Agriculture Organization (FAO) survey, the wheat was cultivated on approximately 150 million hectares and 732.9 million tons was harvested worldwide in 107 countries in 2015 (www.fao.org). Wheat is used both as cash crop and livestock and poultry feed. Being an excellent source of carbohydrate, vitamins, proteins, dietary fibers and minerals, wheat serve as a staple food of many countries and is a major constituent in many foods. The worldwide increasing demand for wheat and wheat products keeps driving the growth of wheat agriculture.

During cultivation, wheat is affected by several diseases such as rusts, smuts, bunts, powdery mildew, leaf blight. Among them, Karnal bunt (KB) incited by smut fungus *Tilletia indica* (Syn. *Neovossia indica*) continues to be a limiting factor in increasing wheat yield. The KB disease (also known as partial bunt) that partially convert the kernels into sori filled with fetid teliospores was first reported in 1931 in Karnal, India [1]. Since then it is widespread in Pakistan, Nepal, Iraq, Iran, Afganistan, South Africa, Mexico and the United States (http://www.nda.agric.za/docs/GenPub/karnalbunt.htm). KB has the potential to reduce the crop yield, seed quality and germination. The wheat lots with 1% or more infected grains make wheat products unpalatable due to unpleasant fishy odor of trimethylamine secreted by teliospores. The wheat containing more than 3% bunted kernels is considered unfit for human consumption [2], as a result the bread making quality wheat becomes downgraded to feed, leading to large financial losses to the producers. The KB disease not only affects the producers but also the wheat exporting countries face the trade barrier due to restricted export and movement of consignment of wheat grains to countries which are presently free from *T*. *indica*. It is now placed as a quarantined pest in more than 70 countries [3]. Moreover, some countries have even imposed zero tolerance quarantine regulations against the KB pathogen by virtue of which wheat importing countries incur indirect costs due to quarantine measures that must be applied to grain exports [4, 5]. Hence, KB is regarded as an economically important disease.

*T*. *indica* is a heterothallic fungi. The teliospores, an infectious entity, are diploid (2N), thick walled, globose or subglobose. Teliospore may remain dormant in soil for several years. On favourable environmental conditions (optimum temperature 15–25°C and relative humidity of more than 82%), teliospores germinate to form promycelium (basidium) that give rise to about 65–185 primary sporidia (basidiospores) [6]. Secondary sporidia formed either from primary sporidia (budding) or from fungal threads of mycelium. During flowering stage, secondary sporidia are dispersed by wind or rain and infects the florets through the ovary wall. Sporidia gain entry into maturing kernels [5] and replace the healthy kernel tissue with sooty mass of teliospores. On germination, primary or secondary sporidia (1N) give rise to monokaryotic hyphae (1N). At an unknown stage, the monokaryotic hyphae with compatible mating types fuse to form dikaryotic hyphae (1N+1N). Within dikaryotic hyphae, compatible nuclei fuse to form teliospores [7].

Despite the economic importance of *T*. *indica*, a meagre knowledge about the molecular components of fungal pathogenesis to cause disease is known. To date, many of the important molecular biology and biochemical studies such as identification of pathogenic determinants or virulence factors could not be carried out in this important fungus as it needs the complete genomic information for elucidation of molecular mechanisms. Since, the genome of *T*. *indica* is small and the genes do not share any high degree of sequence homology even with the closest basidiomycetes fungi (viz. *Ustilago maydis*, *Ustilago hordei*) hampers the development of conserved primers for isolating the target genes. Thus, genome sequencing of the fungus *T*. *indica* is the only alternative to elucidate the pathogenic mechanisms of Karnal bunt of wheat. In this study, the genome of *T*. *indica* is sequenced through hybrid approach using both PacBio Single Molecule Real Time (SMRT) sequencing and Illumina HiSEQ 2000 platform and annotated for identification of pathogenicity genes of *T*. *indica*. This would facilitate identification of biomarkers (pathogenic determinants/virulence factors) that would expedite our efforts towards discovering pathogenicity mechanisms involved in Karnal bunt disease development.

To our knowledge, this is the first draft genome sequence of *T*. *indica* and the first draft genome of wheat bunt fungi. This study would not only serve as model for studying the pathogenic mechanisms in wheat but also allow the identification of the fungal pathogenic determinants/ virulence factors, characterization of signal transduction and biochemical pathways that would be crucial for devising effective crop protection strategies such as development of resistant wheat cultivars through genetic engineering or plant breeding, novel biomarkers for pathogen detection and new targets for fungicide development.

## Materials and methods

### *T*. *indica* isolate and DNA isolation

The highly virulent Karnal (TiK) isolate of *T*. *indica* was used for genome sequencing. The vegetative mycelium of *T*. *indica* was obtained by culturing single germinating teliospore in 3.9% potato dextrose agar (PDA) at 18°C in alternate light and dark conditions. The genomic DNA was isolated using QIAgen DNeasy plant DNA minikit according to the manufacturer’s protocol. The quantity and quality of extracted genomic DNA was determined by spectrophotometry and agarose gel electrophoresis (0.8%), respectively.

### *De novo* genome sequencing and hybrid assembly

Genome of *T*. *indica* Karnal (TiK) isolate was sequenced at NxGenBio Life Sciences, New Delhi India. In concise, the whole genome of *T*. *indica* was *de novo* sequenced through hybrid approach using both Illumina HiSEQ 2000 and PacBio single molecule real time (SMRT) platforms. 100 bp paired end library was prepared using Illumina TruSeq kit following the manufacturer’s instructions and sequenced by Illumina Hiseq 2000 sequencer. Raw reads quality was checked by fastQC (www.bioinformatics.babraham.ac.uk/projects/fastqc/) and the reads with score above 20 were chosen for further assembly. Subsequently, trimming of raw reads was done using fastx toolkit (http://hannonlab.cshl.edu/fastx_toolkit/). Another shotgun library was prepared using PacBio p5c3 chemistry and two SMRT cells were run on Pacbio RSII sequencer. Illumina reads were assembled into contigs using Abyss version 1.3.7. The raw reads of PacBio SMRT were *de novo* assembled by Hierarchical Genome Assembly Process (HGAP) of SMRT Analysis v2.3.0 using default parameters. The contigs of each of Illumina & PacBio SMRT reads were broken with size of 1kb and 500 bp overlap with the previous reads using inhouse developed perl script. These two sets of reads were taken as two input reads for 454 *de novo* Assembly version 2.5.3 to carry out the hybrid assembly.

### Gene prediction and annotation

Quality and completeness of genome was evaluated using Benchmarking Universal Single–Copy Orthologs Version 2 (BUSCO v2) with fungal dataset on all the contigs [8] Gene prediction was performed using Augustus [9, 10, 11] with gene models from *Ustilago maydis* as a reference. Manual inspection of predicted genes was carried out to maximize the accuracy of gene prediction. The protein—coding genes were annotated using NCBI non-redundant (NR), Uniprot, GO, KEGG databases. Matches with e—value ≤ 1xe^-5^ and 40% sequence identity were selected. Genome repetitive elements were analyzed by Blast against the RepeatMasker library (Open 3.2.9) [12].

Non-coding RNA, rRNA and tRNA were predicted by rRNAmmer 1.2 [13] and tRNAscan1.23 [14], respectively.

### Protein family classification

To identify G-protein-coupled receptors, local Blastp search was conducted against GPCRDB database (http://www.gpcr.org/7tm/). Carbohydrate-active enzymes and Protease families were screened through local Blastp search in CAZy [15] and MEROPS peptidase Database [16], respectively. Genes coding for Kinases and Transporters were identified by local Blastp search in KinBase (http://www.kinase.com/) and Transporter Classification Database (www.tcdb.org/), respectively. The candidate virulence associated genes were identified by performing Blastp searches of the *T*.*indica* KBK genome against pathogen- host interaction database (PHI base version 4). The PHI base catalogues experimentally curated pathogenicity, virulence and effector genes from different pathogens (such as oomycete, fungal and bacterial pathogens [17].

### Comparative genome analysis

Predicted genes of *T*. *indica* were compared with the predicted proteins of sequenced fungal genomes such as *U*. *maydis*, *U*. *hordei*, *Sporosorium reilianum*, *Puccinia graminis tritici*, *Saccharomyces cerevisiae*, *Fusarium graminearum*. All proteins were searched against all other proteins in these genomes using BLASTX. Matches with E≤1e^−5^ and at least 30% sequence identity, over 60% of both protein lengths were taken as homologous sequences. Synteny analysis of *T*. *indica* KBK genome was performed with *U*. *maydis*, *U*. *hordei*, *S*. *reilianum* genomes using SyMAPv4.0 program (http://www.symapdb.org/). For phylogenetic analysis, ITS1, 5.8S, ITS2 sequences were used, sequences were retrieved from GenBank (S1 Table). Phylogenetic analysis of *T*. *indica* with other phytopathogenic fungi including *U*. *maydis*, *U*. *hordei*, *S*. *reilianum*, *P*. *graminis tritici* (phylum basidiomycota), *S*. *cerevisiae*, *F*. *graminearum*, *Magnaporthe grisea* (phylum ascomycota) and *Phytophthora infestans* (phylum oomycota) was carried out using MEGA version 7, Maximum Likelihood tree was constructed using Tamura 3-parameter model with 1000 bootstrap replications [18, 19].

## Results and discussion

### *De novo* genome sequencing and hybrid assembly

**Fig 1:**
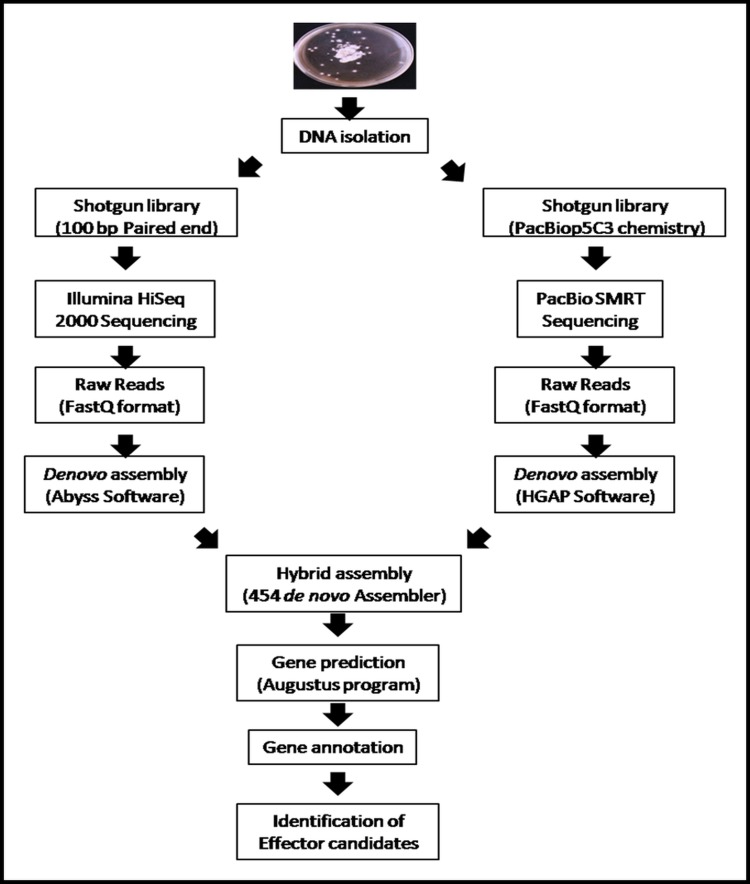
Overall workflow for *de novo* genome sequencing, assembly and annotation of *T*. *indica* Karnal isolate for identification of Effector Candidates.

A good quality high molecular weight DNA fungal gDNA was extracted. The yield of genomic DNA was found to be 316.68μg/50mg of fungal biomass. The whole genome of *T*. *indica* was sequenced by shotgun approach. The overall workflow for *de novo* genome sequencing of *T*. *indica* Karnal isolate is given in Fig 1. A total data of 532304123200 reads were generated by Illumina Hiseq 2000 sequencing that included 5323041232 paired–end reads of 100 bp each. Illumina processed reads were *de novo* assembled into 1125555 contigs using Abyss software. The largest contig size and N50 was 167,021 bp and 5,842, respectively (Table 1).

**Table 1:** Comparison of the Assembly statistics of PacBio SMRT and Illumina HiSeq 2000 Sequencing of the draft genome of *T*. *indica*.

Properties	PacBio SMRT	Illumina HiSeq 2000
Total raw reads	8,45,967	5323041232
Average read length (bp)	16,178	100
Total No. of Contigs	269	11,25,555
Max contig length	14,732	1,67,021
Min contig length	500	500
Mean contig length	3,409	5,269
N50 value Max	3,009	5,842
Sum of Bases in Contigs	1,091,426,619	29,900,000

Another data set was generated using an improved (P5-C3) version of PacBio chemistry and sequenced on two SMRT cells. The P5-C3 chemistry produced a total of 1.19 GB of data. About 8,45,967 reads were generated and had an average length of 16,178 with N50 value of 28,404 (Data not shown). These reads were *de novo* assembled by Hierarchical Genome Assembly Process (HGAP) of SMRT portal into 269 contigs with N50 contig length of 3,009 and maximum contig length of 14,732 (Table 1). The hybrid assembly from both sequencing platforms resulted in 10,957 contigs with 26,707,738 (26.7 Mb) and maximum and N50 contig length of 24,487 and 3,009, respectively (Table 2).

**Table 2:** General features of the assembly statistics of hybrid assembly of draft *T*. *indica* genome.

Features	
Number of Reads	1,90,826
Total Contigs	10,957
N50 Contig Length	3,009
Max Contig Length	24,487
Sum of Contig Lengths	26,707,738
Number of bases	1,273,431,014
G+C content	53.99%
Total Coverage	162X
Predicted Genes	11,535
tRNAs	133
rRNAs	160

### Gene prediction and annotation

**Fig 2:**
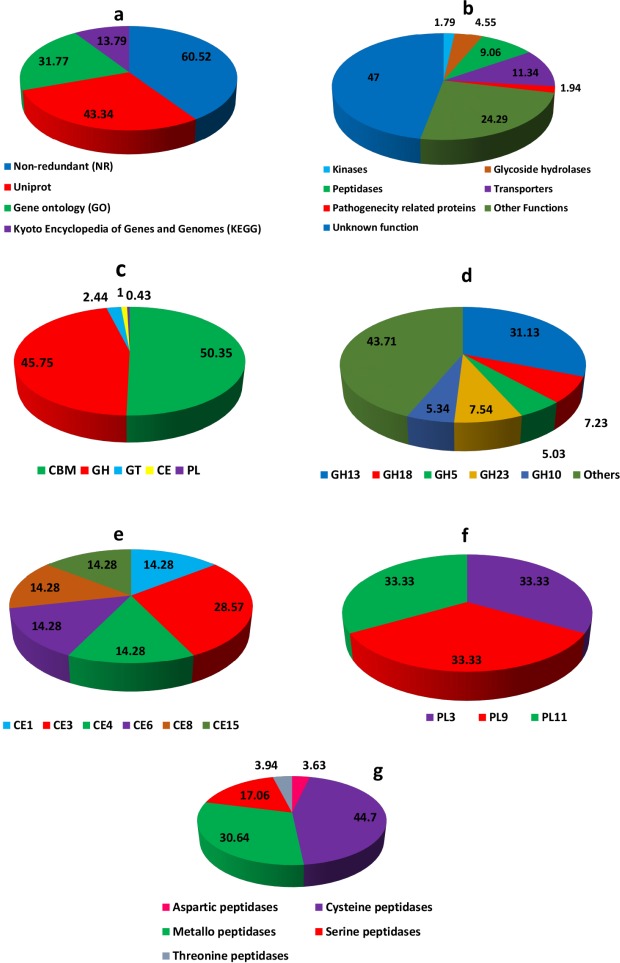
Functional annotation of the T. indica effector candidates (a) Gene annotation of predicted genes using NCBI non-redundant (NR), Uniprot, Gene Ontology (GO), Kyoto Encyclopedia of Genes and Genomes (KEGG) databases. (b) Percentage distribution of the proteins with distinct enzymatic functions. (c) Summary of the CAZyme categories: carbohydrate-binding modules (CBMs), carbohydrate esterases (CEs), glucoside hydrolases (GHs), glycosyl transferases (GTs), polysaccharide lyases (PLs). (d) Percent distributon of most abundant Glycosyl hydrolases (GH). (e) Percent distributon of most abundant carbohydrate esterases (CEs). (f) Percent distributon of most abundant of most abundant polysaccharide lyases (PLs). The prediction of CAZymes from *T*. *indica* effector candidates and their classification were performed using tools from the Carbohydrate-Active EnZymes (CAZyme) database. (g) Percentage distribution of different types of peptidases present in *T*. *indica* genome.

The BUSCO evaluation of completeness of the *T*. *indica* genome sequence predicted that it was 97% complete. A total 1,438 BUSCO groups were searched, The genome assembly found to contain 1,395 complete single-copy BUSCOs, 120 complete duplicated BUSCOs, 13 fragmented BUSCOs, and 25 missing BUSCOs (S2 Table). We predicted 11,535 protein—coding genes which are higher than genes predicted in other basidiomycetes, especially smut fungi. *U*. *maydis* (6,548), *U*. *hordei* (7,110), *S*. *reilianum* (6,673), *S*. *scitamineum* (6,636) [20]. Only 6981 (60.52%), 5000 (43.34%), 3665 (31.77%), 1591 (13.79%) of the predicted genes in *T*. *indica* had homologies with known function in NCBI non-redundant (NR), Uniprot, Gene ontology (GO), Kyoto Encyclopedia of Genes and Genomes (KEGG) databases, respectively. 1591 genes were found to be common to all these protein databases (S3 Table; Fig 2A).

Eukaryotic genome contain huge number of repetitive elements. Repetitive elements comprises 44% of human genome [21] and 75% of maize genome [22]. However, repetitive elements are less abundant in fungal genomes rarely exceeding 5% of the genome [23]. Fungal genomes contain low amounts of repetitive elements due to presence of defense mechanism called repeat- induced point mutation (RIP) [24] that protect fungal genomes against highly repeated sequences. The *T*. *indica* genome was analyzed for repetitive sequences and retro-elements using RepeatMasker 3.2.7 [12], which screens DNA sequences for interspersed repeats and low complexity elements. Simple repeats were the most abundant elements identified by RepeatMasker, totaling 471.7 kb or 1.77% of the genome. There were 1565 low complexity elements identified of total length of 87.0 kb, covering 0.33% of the genome. In total, repetitive elements comprises 3.11% of *T*. *indica* genome (S4 Table). This percentage is consistent with the frequency of repeat elements observed in other fungi, which rarely exceeds 5% of the genome [23].

**Fig 3:**
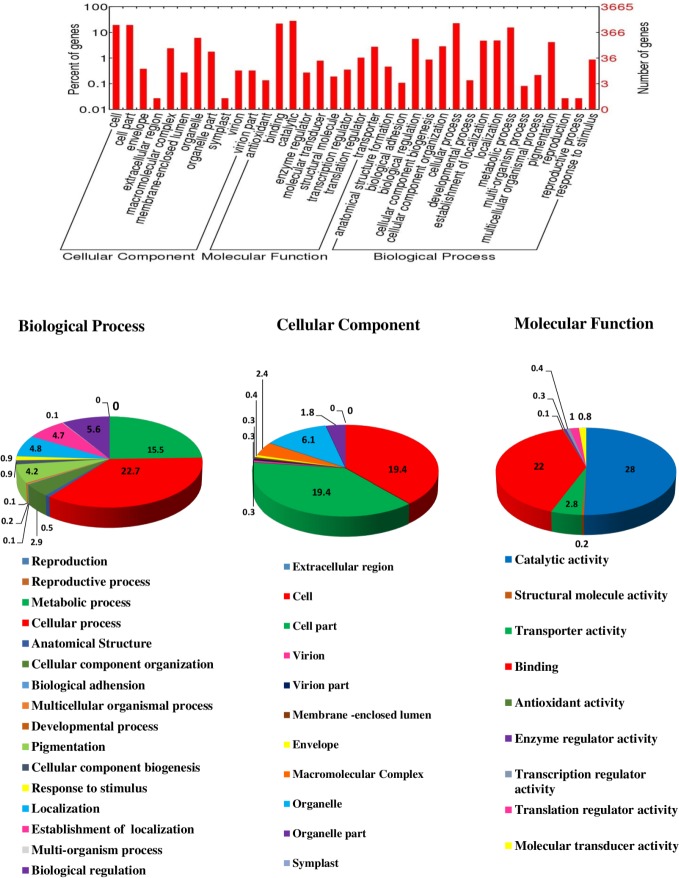
Assignment of Gene ontology (GO) term for predicted genes of *T*. *indica* genome into three categories: cellular component, molecular function and biological process.

The predicted genes were annotated and assigned Gene ontology (GO) terms for three categories as biological process, cellular component and molecular function (Fig 3). A total of 3665 (31.77%) genes in *T*. *indica* genome were assigned GO term. Most of the genes i.e. 2491 (21.59%) genes were assigned GO term for biological process followed by 2037 (17.65%) and 2634 (22.83%) for molecular function and cellular component. In the biological process category, the cellular process (22.7%) and metabolic process (15.5%) were most highly represented in hybrid genome assembly. Amongst the cellular component category, cell (19.4%) and cellular components (19.4%) were dominant. While, catalytic (28%) and binding activity (22%) were most dominant in molecular function category. The effector candidate genes (such as transporter, hydrolase, kinase) were also identified through GO annotations.

### Protein family classification

Data obtained from sequencing of genomes allows to better understand evolution, especially rapidly evolving diversity in fungi. In our study, we observed that the genome of *T*. *indica* is composed of a large number of genes with multiple domain proteins. In a view, a domain can be viewed as a primary unit of evolution [25]. We classified the *T*. *indica* proteome into different protein families based on sequence similarity. In rapidly evolving genomes such as fungi, not all positions in coding regions are equally susceptible to mutations, as some positions may have lower importance. For example, in *T*. *indica* we observed that a significant number of genes identified belonging to functions vital for pathogen survival and successful infection. They include signal pathway related proteins (GPCRs, Protein kinases), Cell wall degrading enzymes (CWDEs), ABC transporters, host cell adhesion, transporters, host defence inhibitors (such as peptidases) and pathogenicity–related proteins (Fig 2B).

G-protein coupled receptors (GPCRs), one of the largest families of transmembrane receptors, that transduce extracellular signals by means of heterotrimeric G protein and complex signalling pathways to ultimately regulate gene expression and intracellular responses. They are involved in sensing various extracellular signals, such as pheromones, sugars, amino acids and nitrogen sources [26]. In general, GPCRs have been categorized into six classes, classes A-E. The classes A—C are mainly present in animals, comprising the rhodopsin, secretin and metabotropic glutamate/pheromone receptors, respectively [26]. The Class D, exclusively present in fungi, has pheromone receptors, class E comprises cAMP receptors and class F includes frizzled/smoothened [26, 27]. A large number of GPCRs (more than 800 members) have been identified in human genome [28]. While few GPCRs have been reported in fungal genomes, as only three and four GPCRs have been reported in the genome of ascomycete *S*. *cerevisiae* and *Schizosaccharomyces pombe* [29, 30]. However, 84 and 61 GPCRs have been reported in head blight fungus *F*. *graminearum* [31] and rice blast pathogen *Magnaporthe grisea* [32], respectively. Five GPCRs have been identified in *T*. *indica* genome, that are grouped into four classes (Table 3). Pth 11, GPCR in *M*. *grisea*, mediates appressorium differentiation and virulence [26]. Like other smut fungi, *U*. *maydis*, *U*. *hordei*, *S*. *reilianum*, *S*. *scitamineum*, the genome of *T*.*indica* does not contains Pth 11- like GPCR (Table 3).

**Table 3:** GPCR in different fungal genomes *.

Classes	*T*. *indica*	*U*. *maydis*	*U*. *hordei*	*S*. *relianum*	*S*. *scitamenium*	*P*. *graminis tritici*	*M*. *oryzae*	*F*. *graminearum*	*V*. *dahliae*
Class I	0	0	0	0	0	0	1	1	1
Class II	1	1	1	1	0	3	1	1	0
Class III	0	0	1	1	1	3	5	2	3
Class IV	1	1	3	1	1	2	2	2	2
Class V	0	0	0	0	0	0	2	4	2
Class VI	0	0	0	0	0	0	2	0	1
Class VII	0	0	0	0	0	0	1	0	1
Class VIII	0	0	0	0	0	2	3	1	2
Class IX	2	2	2	2	2	1	1	3	2
Class X	1	1	1	1	1	1	1	1	1
Class XI	0	0	1	0	0	0	0	0	1
Class XII	0	0	0	0	0	0	2	1	1
Class XIII	0	0	1	1	1	5	2	1	0
PTH 11 like	0	0	0	0	0	0	9	2	3

* Information for fungal genomes (except *T*. *indica*) adapted from Que *et al*, 2014 [20].

Similarly, three main protein kinases mediated signaling pathways (MAPK, cAMP and Ca) govern pathogenicity and infection–related development of the pathogenic fungi. The main groups of fungal protein kinases are STE, CMGC, AGC and CAMK. STE and CMGC kinases have a role in MAPK pathway, AGC is invoved in both cAMP and Ca signaling pathway and CAMK in only Ca signaling pathway [33]. The *T*. *indica* genome contains 125 protein kinases which is fewer than other hemibiotrophic fungi, 147 in *Curvularia lunata* and 140 in *Bipolaris maydis* [33]. In *S*. *cerevisae*, there are five MAPK cascades namely Fus3, Kss1, Hog1, Mpk1 and Smk1 that regulate filamentous growth, pheromone response, hyperosmoregulation and cell wall integrity [33]. The blastp search against KinBase database revealed that the homologues of MAPK pathways of *S*. *cerevisae* are encoded by *T*. *indica* genome (S5 Table). The homologues of MAP kinase (TiFus3, TiPmk1, TiKpp2) were cloned and characterized in our laboratory. Further, these cloned genes were investigated using bioinformatics tools to define their role in fungal pathogenesis [34].

The plant cell wall is composed mainly of polysaccharides pectins, cellulose, hemicellulose, lignin [35]. The lignocellulose degradation requires diverse groups of enzymes such as cellulases, hemicellulose and lignin degrading enzymes. Therefore, plant pathogenic fungi secrete a wide assortment of Carbohydrate degrading enzymes (CAZymes) to breach plant cell wall, penetration and successful infection. Presently, the CAZymes are divided into five families as glycoside hydrolases (GHs) family, glycosyltransferases (GTs) family, polysaccharide lyases (PLs) family, and carbohydrate esterases (CEs) family, based on their structurally—related catalytic modules and functional domains [36]. Due to their vital role in degrading plant biomass, the CAZymes belonging to families GH, CE and PL can also serve as cell wall degrading enzymes (CWDEs) [37, 38]. The CAZyme families such as GH28, GH78, GH88, CE8, PL1-3, PL9 and PL10 utilizes their pectin esterase, pectate lyase, pectin lyase and polygalacturonase (PGA) activity to degrade pectin [36]. The members of GH 28 family with polygalacturonase (PGA) activity can hydrolyze the α-1,4 glycosidic bonds between galacturonic acid residues, a major component of polygalacturonan, which is an important carbohydrate component of the pectin network of plant cell walls. A large family of PGAs have been identified in *Sclerotinia sclerotiorum*, *Botryotinia fuckeliana* and *Rhizopus oryzae* [38]. On the other hand, gene encoding PGA has not been identified in class Saccharomycetes and Schizosaccharomycetes. However, one PGA gene is present in yeast *S*. *cerevisiae* [38]. The CE8 family is homologous to pectinesterases that catalyze the deesterification of pectin to pectate and methanol and play an ancillary role in pectin degradation [38]. *T*. *indica* possess 4 members of GH78 family and 1 member of each CE8 and PL9 (S6 Table). It lacks enzymes belonging to PL1, PL2, PL3, PL10, GH28 and GH88 families. Amongst all CAZymes, the GH class consists maximum number of enzymes involved in lignocellulose degradation. The complete degradation of plant cell wall requires the synergistic action of several enzymes such as xylanases in families GH10, GH3, GH11 and GH39, cellulases in families GH3, GH5, GH45, GH74 and accessory enzymes (e.g. β-galactosidase, α-L- arabinofuranosidase, β-1,4- galactanase etc) in families GH27, GH35, GH54, GH62 [38]. In *T*. *indica* genome, most of the genes (i.e. 24) belongs to GH18 family that possess chitinase activity (EC 3.2.1.14) followed by 16 from GH5 family and 14 each from GH10 family (S6 Table). The protein product of GH5 genes possess β-1,3-glucanase, β-mannanase, and hydrolyze both β-mannans and β-glucans. The GH10 family genes with endo-1,4-β-xylanase (EC 3.2.1.8) can degrade the beta-1,4-xylan into xylose, thus involves in breaking down hemicellulose, a major component of plant cell wall [38]. In all, *T*.*indica* genome encodes 328 putative CAZymes including 350 Carbohydrate binding modules (CBM), 318 Glycoside hydrolases (GH), 17 Glycosyltransferases (GT), 7 Carbohydrate esterases (CE) and 3 Polysaccharide lyases (PL) (S6 Table; Fig 2C, 2D, 2E and 2F).

The *T*. *indica* genome sequenced in this study contains 41 families of proteases and most of them were included in families of cysteine peptidases (283) and metallopeptidases (194) (S7 Table, Fig 2G). Gene expansions within the papain and calpain are consistent with their being virulence factors in plant pathogens. Compared to other plant pathogenic fungi, the S01 trypsin and S16 Lon A peptidase subfamilies are larger and lacks the S08 subtilisin subfamily. The *T*. *indica* genome has 36 trypsin genes compared to 3 or less in other plant pathogenic fungi. Interestingly, C14 caspase and M14 carboxypeptidase is quite higher than other pathogens (S7 Table). The A01 aspartyl proteases serve as virulence factors of plant pathogens due to their ability to cleave a wide range of host proteins [39]. Compared to plant pathogenic fungi (average 18) [39], their number is significantly expanded in the *T*. *indica* genome (average 23) (S7 Table).

One of the largest superfamilies of fungal transporters, ATP-binding cassette (ABC) transporters, facilitate ATP- dependent transport of a wide array of molecules across cellular membrane [40]. ABC transporters contribute to fungal virulence by facilitating the secretion of fungal virulence factors (e.g. toxins, secondary metabolites) and protecting the fungi against plant defense compounds (e.g. host derives antimicrobial compounds such as phytoalexins) [41]. The *T*. *indica* genome encodes 792 transporters with 35 ABC transporters which is close to 37 in *U*. *maydis* but higher than *S*. *cerevisiae* (S8 Table). Within the ABC transporters, pleiotrophic drug resistance (PDR) and multidrug resistance (MDR) subfamilies provide resistance against various antifungal agents [42]. *T*. *indica* has 5 members of MDR transporters subfamily and lack any PDR transporter. Apart from ABC transporters, the transporters of the major facilitator superfamily (MFS) plays a crucial role in overcoming host defense. 13 MFS genes have been identified in *T*. *indica* genome (S8 Table).

The candidate pathogenicity proteins were identified by interrogating *T*. *indica* genome with pathogenicity proteins from the pathogen host interaction (PHI) database. About 135 *T*. *indica* proteins showed homology with pathogenicity proteins of PHI database (S9 Table). Some of the important pathogenicity—related genes includes proteins involved in signaling, surface attachment, RXLR related avirulence effector, Kazal type serine proteases, glucanase inhibitors, necrosis induction, triggering effector immunity against host plant defense molecules, induction of hyper sensitive reaction and death in host plant. Some of these pathogenicity genes could play a vital role in KB disease development in wheat. Further, such pathogenicity genes should be functionally analyzed for unraveling pathogenic mechanisms used by the causal agent (*T*. *indica*) of Karnal bunt disease in wheat.

### Comparative genome analysis

**Fig 4:**
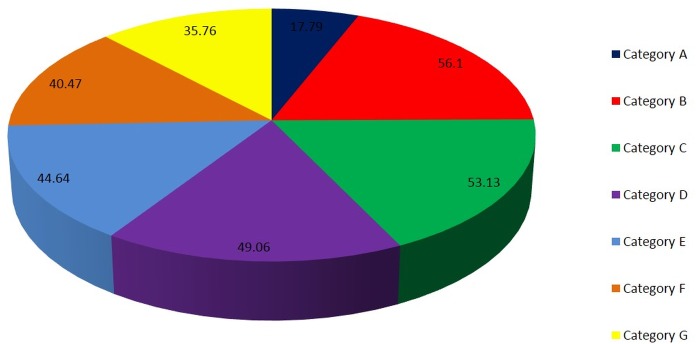
Classification of *T*. *indica* genes into 7 Categories based on Orthology analysis: Category A (only found in *T*. *indica)*, Category B (found in both *T*. *indica* and *U*. *maydis*), Category C (only found in both *T*. *indica* and *U*. *hordei*), Category D (found in both *T*. *indica* and *S*. *reilianum*), Category E (found in both *T*. *indica* and *S*. *cerevisiae*), Category F (found in both *T*. *indica* and *P*. *graminis tritici*), Category G (found in both *T*. *indica* and *F*. *graminearum*).

**Fig 5:**
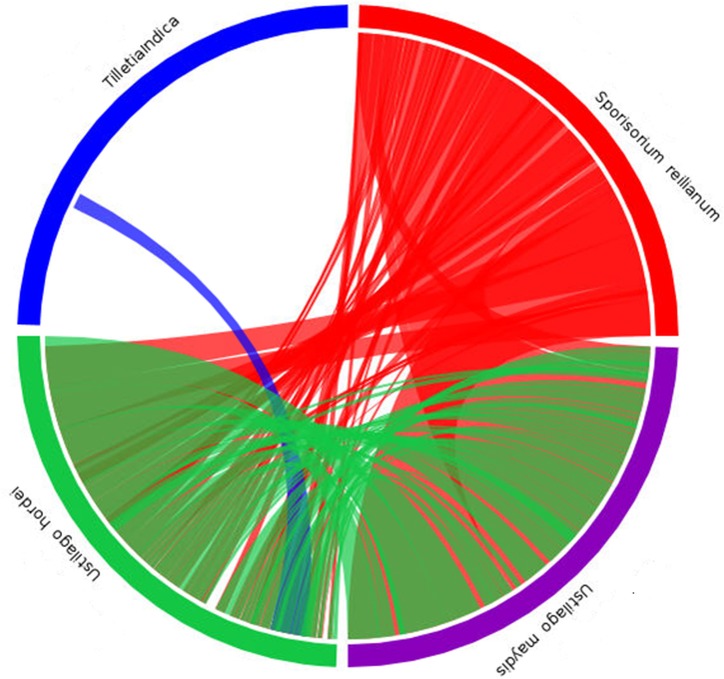
Synteny analysis of *T*. *indica* genome with other smut fungi, *U*. *maydis*, *U*. *hordei* and *S*. *reilianum*.

The orthologous genes between *T*. *indica* and other fungal genomes were identified based on bidirectional best hits (BBHs) using BLAST [43]. On the basis of orthology analysis, the genes in *T*. *indica* can be classified into 7 categories: Category A (only found in *T*. *indica*, 2053 genes), Category B (found in both *T*. *indica* and *U*. *maydis*, 6472 genes), Category C (only found in both *T*. *indica* and *U*. *hordei*, 6129 genes), Category D (found in both *T*. *indica* and *S*. *reilianum*, 5660 genes), Category E (found in both *T*. *indica* and *S*. *cerevisiae*, 5150 genes), Category F (found in both *T*. *indica* and *P*. *graminis tritici*, 4669 genes), Category G (found in both *T*. *indica* and *F*. *graminearum*, 4126 genes) (Fig 4). The results showed that *T*. *indica* shared many genes with other basidiomycetes (*U*. *maydis*, *U*. *hordei*, *S*. *reilianum*) than with other class of fungi. Synteny analysis of *de novo* assembled genome of *T*. *indica* was carried out with *U*. *maydis*, *U*. *hordei* and *S*. *reilianium*. Only a small portion of the assembled sequence of *T*. *indica* showed synteny with *U*. *hordei*. No synteny was observed with the other two genomes (Fig 5).

**Fig 6:**
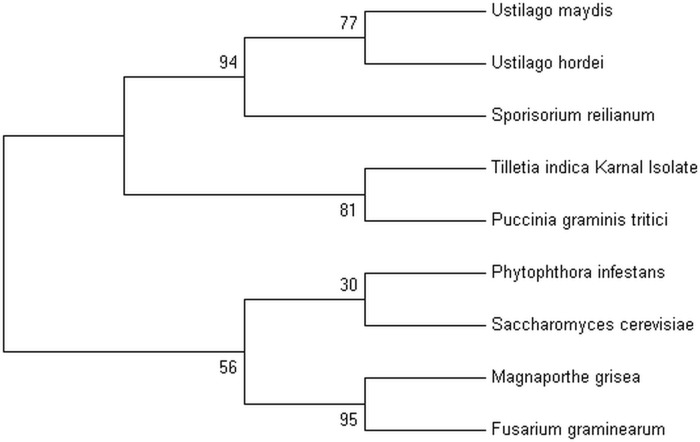
Phylogenetic tree showing relationship of *T*. *indica* genome with genomes of other phytopathogenic fungi. Maximum Likelihood tree was constructed using ITS1, 5.8S, ITS2 sequences using MEGA version 7, (Tamura 3-parameter model) with 1000 bootstraps.

Phylogenetic analysis was conducted to determine the evolutionary relationship of *T*. *indica* with other phytopathogenic fungi including *U*. *maydis*, *U*. *hordei*, *S*. *reilianum*, *P*. *graminis tritici* (phylum basidiomycota), *S*. *cerevisiae*, *F*. *graminearum*, *M*. *grisea* (phylum ascomycota) and *P*. *infestans* (phylum oomycota). The phylogeny revealed two clades—all the members of the phylum basidiomycota including *T*. *indica* grouped together in one clade while the other clade consisted the members of the phylums ascomycota and oomycota. Interestingly, *T*. *indica* Karnal isolate showed close resemblance with *Puccinia graminis tritici*. Members of the genera Ustilago and Sporisorium seem to be diverged from the *T*. *indica* and *P*. *graminis tritici* (Fig 6). The members of the phylum basidiomycota seem to have diverged from the members of ascomycota and oomycota. It was of utmost surprise that *T*. *indica* Karnal isolate showed close relatedness with *P*. *graminis tritici*. However, it also indicates molecular evolution with other members of basidiomycetes including *Ustilago* and *Sporisorium*.

## Conclusion

Although, Karnal bunt was first reported in 1931, knowledge about its pathogenic mechanisms is still at its infancy. In this study, we present the draft genome sequence of *T*. *indica*, an economically important quarantine fungal pathogen of wheat. The *T*. *indica* genome sequence and annotation have unearth a treasure trove of genes and pathways involved in pathogenicity such as GPCRs, Carbohydrate active enzymes especially Cell Wall Degrading Enzymes (CWDE), Peptidases, Kinases, Transporters. Moreover, protein family classification based on protein sequence features have enabled to define more accurately the functions of all the potential pathogenic determinants, especially virulence- associated genes that needs to be validated. 135 candidate pathogenicity genes were identified by interrogating the *T*. *indica* genome with pathogen host interaction database. Some pathogenicity—related genes includes proteins involved in signaling, surface attachment, RXLR related avirulence effector, Kazal type serine proteases, glucanase inhibitors, necrosis induction, triggering effector immunity against host plant defense molecules, induction of hyper sensitive reaction and death in host plant. They may have an important role in KB disease development. The comparative genome and phylogenetic analysis with other phytopathogenic fungi have contributed to our understanding of its close evolutionary relationship with related smut fungi. Further, sequencing of *T*. *indica* from different geographical locations will lead to comprehensive evolutionary and population genetic study. In summary, in the present study, we have obtained the draft genome sequence of *T*. *indica* by employing the hybrid approach of PacBio SMRT and Illumina HiSEQ 2000 sequencing and annotated for identifying candidate effector genes for unraveling the pathogenic mechanisms used by economically important quarantine pathogen, *T*. *indica*. Genome sequence reported here would facilitate the researchers in devising effective crop protection strategies such as novel biomarkers for pathogen detection, development of resistant wheat cultivars through genetic engineering or plant breeding and new targets for fungicide development.

## Supporting information

S1 Table Accession number of ITS1, 5.8S, ITS2 sequences of related phytopathogenic fungi, retrieved from GenBank. (XLSX)

S2 Table Assessment of *T*. *indica* genome quality by BUSCO. (XLSX)

S3 Table Protein database annotation results for proteins identified in the genome of *T*. *indica*. (XLSX)

S4 Table Repeat elements in the *T*. *indica* genome. (XLSX)

S5 Table Kinase families in *T*.*indica* genome, classed by KinBase database. (XLSX)

S6 Table CAZymes in *T*.*indica* genome, classed by CAZy database. (XLSX)

S7 Table Proteases in *T*.*indica* genome, classed by MEROPS peptidase database. (XLSX)

S8 Table Transporters in *T*. *indica* genome, classed by Transporter Classification database. (XLSX)

S9 Table Identification of candidate pathogenicity genes through interrogation of the *T*.*indica* genome with the PHI database. (XLSX)
